# PD-L1 May Mediate T-Cell Exhaustion in a Case of Early Diffuse Leishmaniasis Caused by *Leishmania* (L.) *amazonensis*

**DOI:** 10.3389/fimmu.2018.01021

**Published:** 2018-05-11

**Authors:** Daniel Holanda Barroso, Sarah De Athayde Couto Falcão, Jorgeth de Oliveira Carneiro da Motta, Laís Sevilha dos Santos, Gustavo Henrique Soares Takano, Ciro Martins Gomes, Cecília Beatriz Fiuza Favali, Beatriz Dolabela de Lima, Raimunda Nonata Ribeiro Sampaio

**Affiliations:** ^1^Dermatomicology Laboratory, Faculty of Medicine, University of Brasília, Brasília, Brazil; ^2^Post-Graduate Program in Health Sciences, Faculty of Health Sciences, University of Brasília, Brasília, Brazil; ^3^Department of Cell Biology, Institute of Biological Sciences, University of Brasília, Brasília, Brazil; ^4^Tropical Medicine Nucleus, Faculty of Medicine, University of Brasília, Brasília, Brazil; ^5^Internal Medicine Department – Dermatology Service, Faculty of Medicine, University of Brasília, Brasília, Brazil; ^6^Post-Graduate Program in Medicine, Faculty of Medicine, University of Brasília, Brasília, Brazil; ^7^Pathology Service, Hospital Universitário de Brasília, Brasília, Brazil; ^8^Post-Graduate Program in Microbial Biology, Institute of Biological Sciences, University of Brasília, Brasília, Brazil

**Keywords:** diffuse cutaneous leishmaniasis, programmed cell death-1 ligand 1, *Leishmania* (L.) *amazonensis*, interferon-gamma, granzyme B, T-cell exhaustion

## Abstract

**Introduction:**

Diffuse cutaneous leishmaniasis (DCL) is a rare disease form associated with *Leishmania* (L.) *amazonensis* in South America. It represents the “anergic” pole of American Tegumentary Leishmaniasis, and the explanation for its resistance to treatment remains elusive. We aimed to study some possible immunological mechanisms involved in the poor DCL treatment response by evaluating some cell surface molecules obtained from a patient with DCL by flow cytometry.

**Case presentation:**

A 65-year-old DCL patient who initially failed to respond to the standard treatment for the disease showed vacuolated macrophages filled with amastigotes in lesion biopsy, and *L*. (L.) *amazonensis* was identified through ITS1PCR amplification. The *Leishmania* skin test and indirect immunofluorescence analysis revealed negative results. Peripheral blood from the patient was collected after a few months of treatment, when the patient presented with no lesion. Peripheral blood mononuclear cells were analyzed *ex vivo* and *in vitro* after 48 h of stimulation with soluble *L*. (L.) *amazonensis* antigen (SLA). Cell death, surface molecules, and intracellular molecules, such as IFN-γ and granzyme B, were analyzed in the cells using flow cytometry. Analysis of the surface markers showed an increased expression of the inhibitory molecule programmed death ligand 1 (PD-L1) in the monocytes restimulated with SLA (approximately 65%), whereas the negative controls were 35% positive for PD-L1. Conversely, compared with the negative controls, we observed a decrease in CD4^+^IFN-γ^+^ T cells (8.32 versus 1.7%) and CD8^+^IFN-γ^+^ T cells (14% versus 1%). We also observed a relevant decrease in the granzyme B levels in the CD8^+^ T cells, from 31% in the negative controls to 5% after SLA restimulation.

**Conclusion:**

The dysfunctional activation of PD-L1 inhibitory pathway after *Leishmania* antigen stimulation and reduced levels of IFN-gamma and granzyme B-producing cells could be closely related to unresponssiveness to standard drug treatment of DCL patient.

## Introduction

Even though Leishmaniasis is an important health problem, with an estimated incidence of 0.9–1.6 million each year, it is often overlooked during discussions of tropical diseases (1, 2). It is a complex zoonotic disease, with multiple animal reservoirs, and is primarily transmitted by the sandfly host (1). There is no human vaccine, and the treatment available today is based on toxic and poorly tolerated drugs (3, 4). The spectrum of clinical presentations can be divided into the following four categories: cutaneous leishmaniasis (CL), diffuse CL (DCL), mucocutaneous leishmaniasis (MCL), and visceral leishmaniasis (5). The form and severity of infection are related to the infecting *Leishmania* species, together with the host immune response (5, 6). DCL is a rare disease that affects patients showing an impaired cellular immune response against the parasite (7). *Leishmania* (L.) *amazonensis* is the primary species associated with this disease in South America. The disease is characterized by the presence of nodules, plaques, and, in some cases, ulcerated lesions. Histopathological examinations show an abundance of *Leishmania*-infected macrophages that fail to control the infection. The treatment is frequently ineffective, and patients are administered several toxic therapies; however, they experience poor clinical responses (7). The molecular mechanisms regulating the relationship between the parasite and the affected patient are not totally understood. DCL patients frequently show impairment of the Th1/cellular response; however, it is unclear why patients show a poor response to treatment (8).

This case report describes the flow cytometric analysis of the cell expression profile of surface and intracellular molecules from a DCL patient who presented with clinical relapse following standard treatment with *N*-methyl glucamine (NMG). The study aimed to identify the possible immunological mechanisms that lead to poor responses to DCL treatment.

## Case Report

**Figure 1:**
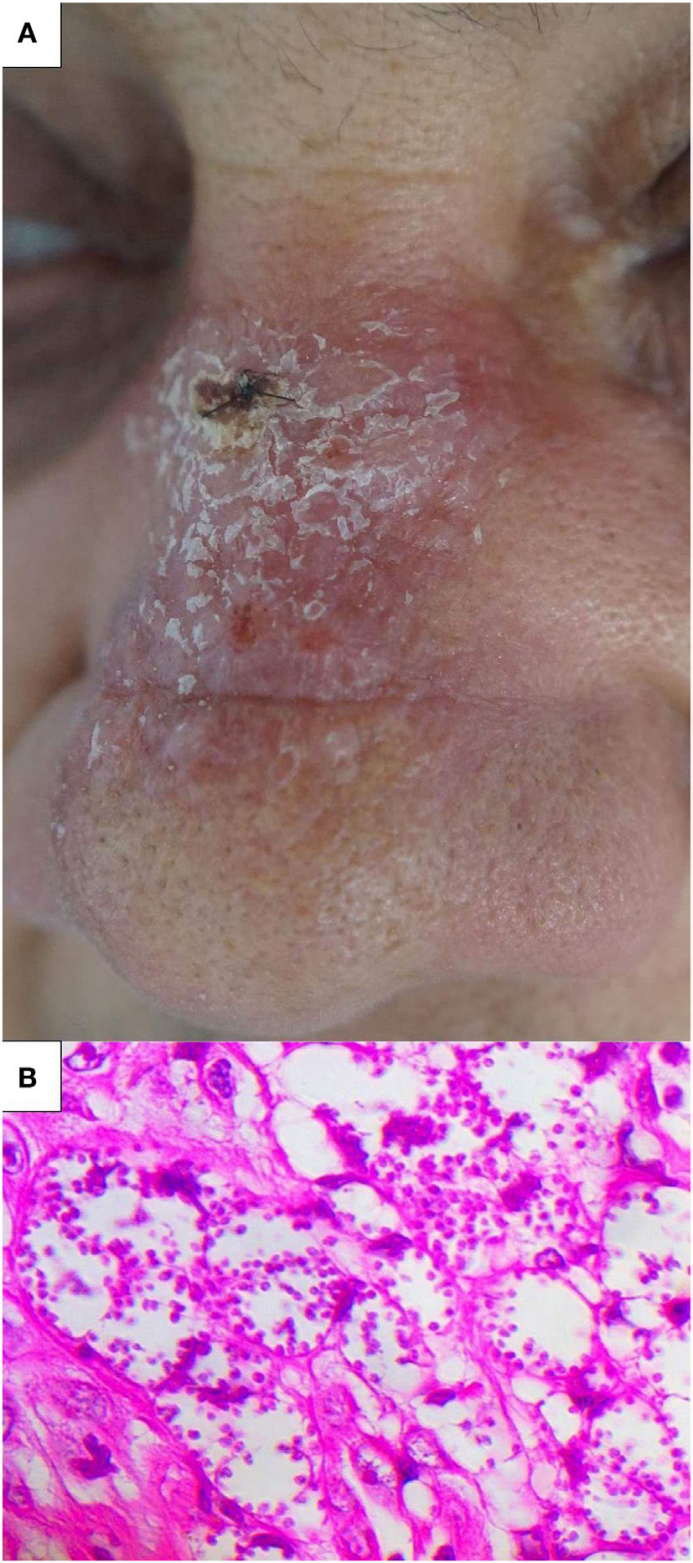
Clinical presentation. **(A)** Erythematous plaque on the nose and **(B)** skin biopsy showing vacuolated macrophages with abundant parasites (HE, 400×).

A male 65-year-old resident of Brasília, Federal District, Brazil, presented with an erythematous plaque on his nose one month after fishing in the state of Amazonas, Brazil (Figure 1). Samples from the lesion aspirate were inoculated and cultured in McNeal, Novy & Nicolle medium, and smears from the skin biopsies were positive for *Leishmania*. Total DNA from isolated parasites in the culture was purified for molecular analysis using the ITS1 PCR amplification and restriction fragment length polymorphism (RFLP) procedures with the HaeIII restriction enzyme (9), which identified the species as *L*. (L.) *amazonensis*. The *Leishmania* skin test (LST) and indirect immunofluorescence results were negative. Microscopic analysis of biopsy showed vacuolated macrophages filled with amastigote forms. All diagnostic procedures are described elsewhere (7, 10). HIV serology was negative.

**Figure 2:**
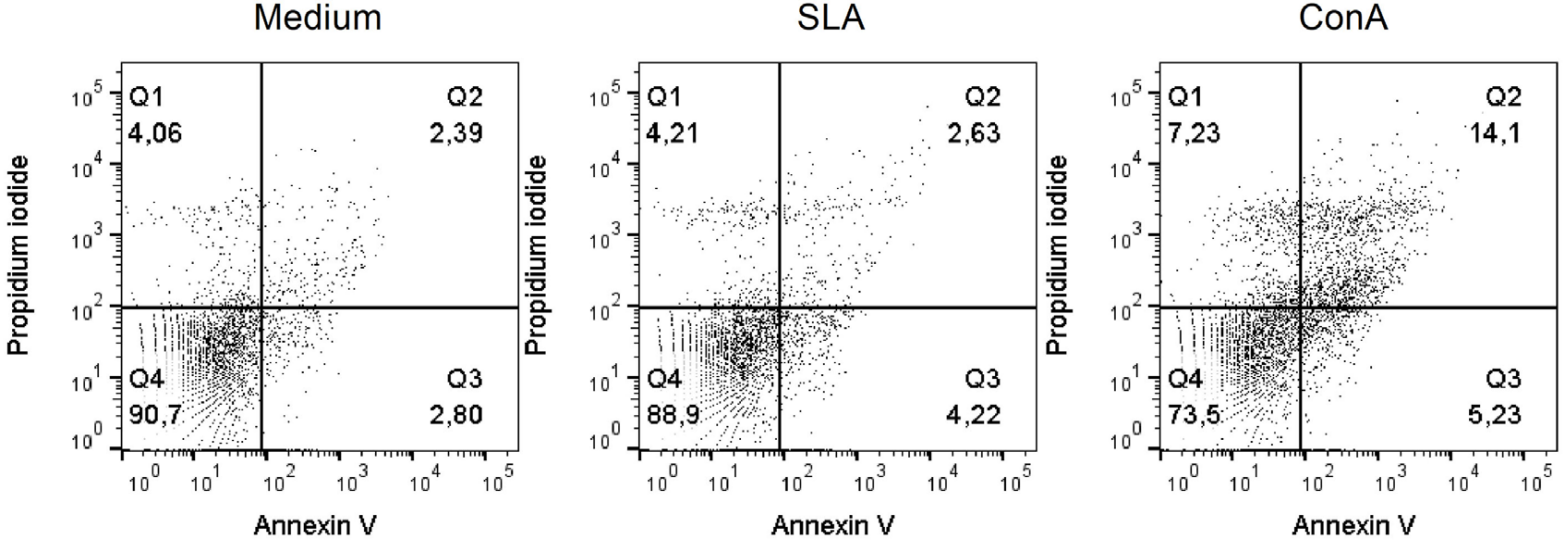
Cellular viability of total peripheral blood mononuclear cells (PBMCs) after SLA stimulation. The patient’s total PBMCs were stained with Annexin V and propidium iodide. Medium (M), soluble *Leishmania amazonensis* antigen (10 µg/mL SLA), and concanavalin A (15 µg/mL conA) after 48 h of *in vitro* restimulation.

**Figure 3:**
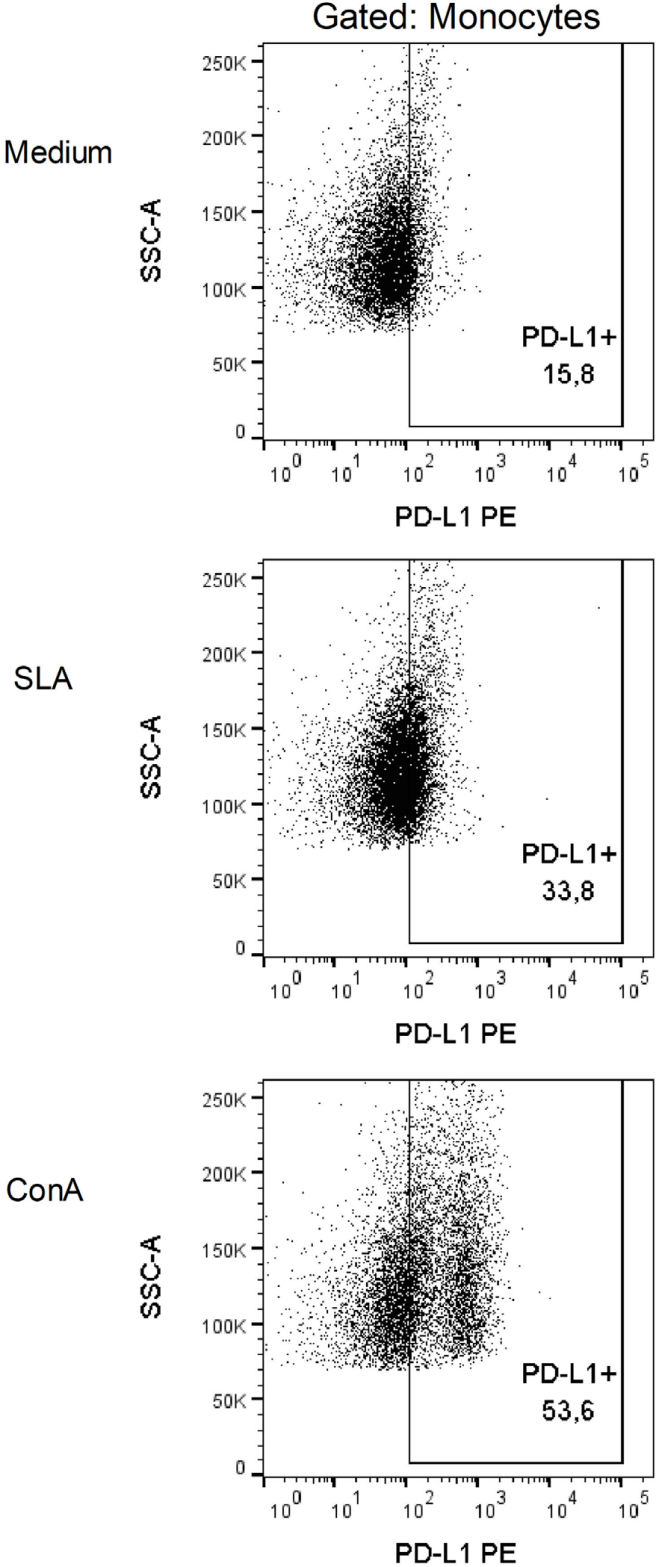
Programmed death ligand 1 (PD-L1) expression in monocytes is increased after SLA restimulation. The patient’s total Peripheral blood mononuclear cells were stained with anti-PD-L1 antibody. PD-L1 expression was analyzed in a population of monocytes gated by size (FSC) and granularity (SSC). Medium (M), soluble *Leishmania amazonensis* antigen (10 µg/mL SLA), and concanavalin A (15 µg/mL conA) after 48 h of *in vitro* restimulation.

**Figure 4:**
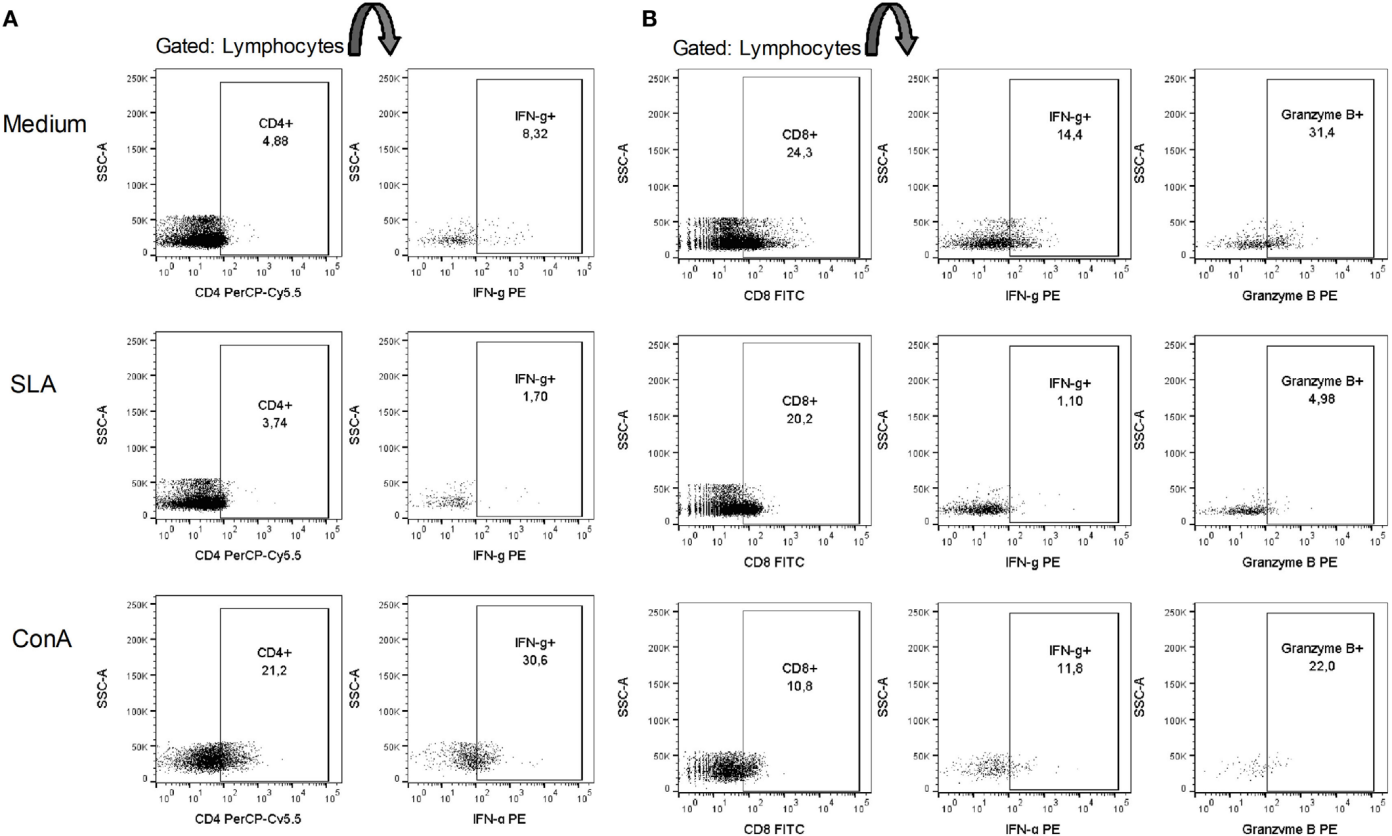
SLA decreased IFN-γ production by CD4^+^ cells and IFN-γ/granzyme B production by CD8^+^ T cells. The patient’s total peripheral blood mononuclear cells were stained with anti-CD4, anti-CD8, anti-IFN-γ and anti-granzyme B antibodies. **(A)** Lymphocytes were gated by size (FSC) and granularity (SSC), and IFN-γ was analyzed in a CD4^+^ population. **(B)** IFN-γ or granzyme B expression was analyzed in a CD8^+^ population. Medium (M), soluble *L. amazonensis* antigen (10 µg/mL SLA), and concanavalin A (15 µg/mL conA) after 48 h of *in vitro* restimulation.

The patient was initially treated with NMG (20 mg SbV/kg/day) for 20 days; however, no improvement was observed (11). The treatment was continued with the administration of 26.775 g that did not result in any improvement. After one month of not undergoing treatment, the patient resumed treatment with 20 mg SbV/kg/day NMG combined with allopurinol (1,200 mg/day). The NMG treatment was stopped after the administration of a 44.625 g dose due to intense myalgia. However, the patient resumed treatment one week later until the total dose reached 53.55 g. We submitted this report 14 months after the administration of the first dose of NMG. The patient was still using allopurinol at that time. To determine the possible causes of this antigen-specific immune dysfunction, we performed *ex vivo* and *in vitro* flow cytometric analysis of the peripheral blood mononuclear cells (PBMCs) of this patient. In this way, after the patient has undergone 11 months of treatment, being only administered allopurinol and presented with no lesion, we collected peripheral blood for immunological analysis. PBMCs were obtained through Ficoll gradient purification and analyzed *ex vivo* and *in vitro* after 48 h of stimulation with soluble *L*. (L.) *amazonensis* antigen (SLA). In *ex vivo* and *in vitro* experiments, the cells were stained with fluorochrome-conjugated antibodies to analyze the surface molecules [CD14, CD40, CD44, CD80, programmed death ligand 1 (PD-L1), HLA-DR, CD86, CD3, CD4, CD8, CD45RO, CD45RA, and CD25]. For the *in vitro* experiment, positive control cells were stimulated with concanavalin A (conA). Cellular viability was assessed using Annexin V and propidium iodide (PI) labeling, acquired by flow cytometry (Verse/BD Biosciences, San Jose, CA, USA) and analyzed using FlowJo software (FlowJo, Ashland, OR, USA). To evaluate the production of intracellular molecules (IFN-γ and granzyme B), Brefeldin A was added to stop protein secretion 4 h before the staining and observation by flow cytometry. The *ex vivo* (SLA unstimulated) experiments showed that the monocytes expressed higher percentages of CD14, CD40, CD44, HLA-DR, and CD86 and lower percentages of CD80 and PD-L1 (Table 1). The *in vitro* experiments showed that the cells were viable, and no differences in the cell death markers were found between the SLA-treated cells and negative controls (unstimulated) (Figure 2). We observed an increase in the expression of the inhibitory molecule PD-L1 in the monocytes restimulated *in vitro* with SLA (approximately 65%), whereas the negative controls were 35% positive for PD-L1 (Figure 3). Additionally, 8.32% of the negative control cells were CD4^+^IFN-γ^+^, but this percentage decreased to 1.7% after SLA stimulation. Analysis of the negative controls showed that 14% of the cells were CD8^+^IFN-γ^+^, but this percentage decreased to 1% after SLA restimulation. We also observed a relevant decrease in granzyme B expression in CD8+ T cells, from 31% in the negative controls to 5% after SLA restimulation (Figure 4).

**Table 1:** Surface molecule expression in *ex vivo* and *in vitro* monocytes.

Surface molecules	*Ex vivo*	*In vitro*		
		Medium	SLA	ConA
CD14	84.9	98.3	96	59.6
CD44	99.3	99.4	99.5	97.6
HLA-DR	88.5	86.4	94.8	25.6
CD86	90.4	78.7	81.2	5.22
CD80	0.46	25.2	32.5	5.2
CD40	91.3	97.1	97.4	71.9
Programmed death ligand 1 (PD-L1)	1.19	35.5	64.9	43.2

*The patient’s total peripheral blood mononuclear cells were stained with specific antibodies against monocyte surface molecules. Surface molecule expression was analyzed in a population of monocytes gated by size (FSC) and granularity (SSC)*.

## Discussion

Our patient, contrary to the expected, after complete clinical response to treatment, more than a year after the disease onset and with no lesion, presented a decrease in CD4+IFN-γ+ and CD8+IFN-γ+ after SLA restimulation. Data from literature show that a classical Th1 cytokine, IFN-γ, plays diverse roles and has different patterns of expression, depending on the infecting *Leishmania* species. In humans, *L*. (L.) *amazonensis* antigen is a weaker inducer of IFN-γ production by CD4+ T cell compared with *Leishmania* (V.) *braziliensis* antigen *in vitro* (12). In contrast, *in vitro* production of IFN-γ by PBMCs is the best indicator of specific immunogenicity as it has been an indicator of response to vaccine against dermotropic *Leishmania* species in humans (13). Nevertheless, IFN-γ plays a paradoxical role during the *L*. (*L*.) *amazonensis* infection. It favors antigen presentation and serves as an inducer of effector mechanisms, particularly through inducible nitric oxide synthase expression; however, it also induces chemokine expression, which leads to migration of CD4+ and macrophages to lesion sites, which are important in disease development (14).

These apparently contradictory findings may be explained by the chronology this cytokine production in the disease course and the *Leishmania* species studied. A human study in a *L*. (V.) *braziliensis* area showed that despite the low or absent IFN-γ production early in the disease, all patients with more than 60 days of cutaneous ulcers presented a strong antigen-stimulated production of IFN-γ by PBMCs (15). Regarding the effect of treatment in IFN-γ responses by human PBMCs, although one study has shown a tendency for higher levels after treatment (16) and another showed a tendency for decreased production (15), more recent reports have not shown significant differences between patients during the active disease and after treatment (17, 18). Together, these studies suggest that IFN-γ is an important molecule in the late stages of *Leishmania* infection because it is present after treatment.

Although CD8+ IFN-γ production is generaly protective in Leishmaniasis, it is suggested that their cytolytic activity is associated with pathology (19). An *in vitro* study with PBMCs from patients with localized CL (LCL) and DCL has suggested that cytotoxicity is mediated by cytotoxic granules and that it is associated with granzyme B expression (20). Granzyme B seems to be part of the acquired *Leishmania*-specific immune response (21), and it is more highly expressed in late cutaneous lesions than in early ones (22). However, it is surprising that our patient presented with diminished granzyme B expression after SLA restimulation late in the disease course and with no lesions at that point.

We also observed that the patient PD-L1 expression on monocytes differed between the SLA restimulated and the negative control. Programmed death-1 (PD-1) is an inhibitory receptor that is primarily present on the surface of activated T cells (23), whereas its ligands (PD-L1 or PD-L2) are present on a variety of cell types, including antigen-presenting cells and many non-hematopoietic cells (24). PD-1 acts as a co-inhibitory receptor during the interaction of the antigenic peptide/MHC with the T-cell receptor (TCR) (23, 24). In addition to its role in self-tolerance, PD-1 is linked to pathological antigen-specific CD8+ and CD4+ T-cell dysfunction in chronic infections and parasitic diseases. This process is called cellular exhaustion (23, 25). Exhaustion can lead to a loss in effector function by affecting the IL-2, IFN-γ, and TNF-α production of T cells, cytolytic activity and degranulation of CD8+ T cells (23, 26). Different from anergy, which is rapidly induced during the first encounter with the antigen, exhaustion is progressive over time (27), and the IFN-γ production and capacity to degranulate seems to be lost later, when the process is advanced (27, 28). In the present study, after SLA restimulation, we observed a decrease in IFN-γ expression by CD4+ and CD8+ and granzyme B expression by CD8+. In contrast, PD-L1 expression increased in the presence of SLA. CD8+ T-cell exhaustion has been previously suggested in Diffuse Leishmaniasis caused by *Leishmania* (L.) *mexicana* (20). These results suggest that the increase in the PD-L1 expression observed in this work might be a mechanism that parasites exploit to avoid the immune response. Our findings suggest that PD-L1 expression by monocytes might play a role in CD8+ and CD4+ T-cell dysfunction in DCL. Interestingly, this T-cell dysfunction was observed months after the initiation of the Leishmaniasis treatment, despite the increase of costimulatory molecules after SLA stimulation, and it resulted in low IFN-γ in CD8+ and CD4+ and granzyme B on CD8+. Together it is possible to assume that, as a consequence of the chronic exposition to the *Leishmania* antigen, this patient presented with T-cell exhaustion that persisted despite clinical cure. This is in accordance with the concept that exhaustion takes between 2 and 5 weeks to develop and affects both cytotoxic and helper functions, and exhausted cells do not recover their functions even after the removal of the antigen (28, 29).

Our patient showed a higher percentage of CD8 T cells than CD4 T in the unstimulated condition. One possibility is that the treatment was sufficient, by this time of immunological evaluation, to restore the CD8 T cells numbers and not CD4 T cells. This observation can suggest a different role of CD8 T lymphocytes in our DCL patient. In fact, data from literature show that the number of CD4 and CD8 lymphocytes from DCL patients peripheral blood can ne altered during the healing process, increasing 86.5 and 37%, respectively and normalizing the CD4/CD8 ratio. In a moment prior to treatment, the CD8 T cells from DCL patients were found diffusely distributed by the inflammatory tissue, whereas after the treatment, a higher number of CD8 T lymphocytes was found localized in the inflammatory infiltrate. However, 5 months after the end of the treatment, CD8 T cells decreased to abnormal numbers in the peripheral blood (30). We observed a decrease of IFN-γ and granzyme B in SLA stimulated CD8+ cells. The viable CD8 T cells from lesion of DCL patients caused by *L. mexicana* showed a decrease in cytotoxicity, antigen-specific proliferation and IFN-γ production after co-cultured with macrophages infected with this parasite. This anergic response by CD8 T cells was restored after stimuli by two TLR2 antagonists (LPG and Pam3Cys). Also, PD-1 expression on CD8 T cells was decreased after the stimuli (20). All these results suggest that the low presence of CD4 cells and, mainly, the anergic CD8 cells can be contributing to unresponsitivity to treatment and probably, to the persistence of DCL.

Diffuse CL is associated with difficult therapeutic management and is often a chronic infection (31). PD-1 blockade using monoclonal antibodies is currently under investigation for the treatment of other chronic infectious diseases and has shown promising results (32). Thus, studying this receptor in DCL is a relevant step toward the development of better therapeutic strategies. The patient showed good clinical results after combination treatment with NMG and allopurinol. Early diagnosis and treatment are crucial for achieving a satisfactory clinical outcome. As shown by our results, despite treatment and a patient without lesions at the time of immunological evaluation, a selective exhausted state can persist after *Leishmania* antigen stimulation and this state is possibly caused by the induction of the PD-L1 inhibitory pathway, once we observed a higher expression of PDL-1 in monocytes.

The increase in PD-L1 expression was also observed through recognition of polyI:C by TLR3 in a CD40/CD40L interaction and NFkB-dependent manner (33). Although TLR3 is a receptor that recognizes double-stranded RNA (dsRNA), many studies have identified the presence of *Leishmania* RNA virus (LVR) in several *Leishmania* species, such as *L*. (V.) *braziliensis, Leishmania* (V.) *guyanensis, Leishmania* (L.) *aethiopica, Leishmania* (L.) *major*, and *Leishmania* (L.) *infantum* (33–37). Murine studies have found an increase in cytokine and chemokine production, susceptibility to parasites, footpad swelling and parasitemia after *Leishmania* infection and recognition by TLR3 (38). Moreover, the presence of the LVR in *Leishmania* has been associated with non-response to treatment. In a recent cohort of 76 American Tegumentary Leishmaniasis patients, subjects infected with *L*. (V.) *guyanensis* that carried LVR did not respond to treatment, while subjects with *Leishmania* without the RNA virus did respond to treatment (36). Treatment failure was also observed during *L*. (V.) *braziliensis* with LVR infection (37). A study performed in the state of Rôndonia found an association of mucosal Leishmaniasis and the presence of the RNA virus in *Leishmania* species (39). In the same study, LVR was found in *L*. (L.) *amazonensis* isolated from two patients (39). Although the state of Minas Gerais has a low incidence of LVR in *L*. (V.) *braziliensis*, most prospective studies that identified the presence of LVR1 are from the Amazonian region (40). In this way, it is worth on note that our patient reported having been in the state of Amazonas before the onset of the lesions. *In vitro* experiments showed an increase in PD-L1 expression and the exhaustion of CD4+ and CD8+ T cells after SLA stimulation. Thus, we suggest that LVR is present in *L*. (L.) *amazonensis* and recognized by TLR3, which would trigger the increase in PD-L1 expression, leading to the exhaustion of T cells, as evidenced by the decrease in the expression of IFN-γ and granzyme B.

Based in our *in vitro* experiments results, we suggest that PD-L1 inhibitory pathway may be related to unresponsiveness to standard drug treatment of DCL patient.

## Ethics Statement

Written informed consent was obtained from the patient prior to collect the biological samples. The authors state that they have obtained a written consent from the patient to publish this case report in this journal.

## Author Contributions

DB, SF, CF, BL, and RS conceived and designed the study. SF and BL performed the experiments. DB, SF, CF, LS, BL, and RS contributed to data analysis. LS processed the diagnostic samples to culture and direct examination. DB, JM, GT, CG, and RS contributed to patient clinical managing. CF, BL, and RS provided materials and infrastructural support. DB, SF, CG, CF, BL, and RS wrote and revised the article.

## Conflict of Interest Statement

The authors certify that they have no commercial or financial relationships that could be a potential conflict of interest.
